# Molecular Cloning and Optimization for High Level Expression of Cold-Adapted Serine Protease from Antarctic Yeast *Glaciozyma antarctica* PI12

**DOI:** 10.1155/2014/197938

**Published:** 2014-06-30

**Authors:** Norsyuhada Alias, Mu'adz Ahmad Mazian, Abu Bakar Salleh, Mahiran Basri, Raja Noor Zaliha Raja Abd. Rahman

**Affiliations:** ^1^Enzyme and Microbial Technology Research Centre, Faculty of Biotechnology and Biomolecular Sciences, Universiti Putra Malaysia (UPM), 43400 Serdang, Selangor, Malaysia; ^2^Enzyme and Microbial Technology Research Centre, Faculty of Science, Universiti Putra Malaysia (UPM), 43400 Serdang, Selangor, Malaysia

## Abstract

Psychrophilic basidiomycete yeast, *Glaciozyma antarctica* strain PI12, was shown to be a protease-producer. Isolation of the PI12 protease gene from genomic and mRNA sequences allowed determination of 19 exons and 18 introns. Full-length cDNA of PI12 protease gene was amplified by rapid amplification of cDNA ends (RACE) strategy with an open reading frame (ORF) of 2892 bp, coded for 963 amino acids. PI12 protease showed low homology with the subtilisin-like protease from fungus *Rhodosporidium toruloides* (42% identity) and no homology to other psychrophilic proteases. The gene encoding mature PI12 protease was cloned into *Pichia pastoris* expression vector, pPIC9, and positioned under the induction of methanol-alcohol oxidase (*AOX*) promoter. The recombinant PI12 protease was efficiently secreted into the culture medium driven by the *Saccharomyces cerevisiae* *α*-factor signal sequence. The highest protease production (28.3 U/ml) was obtained from *P. pastoris* GS115 host (GpPro2) at 20°C after 72 hours of postinduction time with 0.5% (v/v) of methanol inducer. The expressed protein was detected by SDS-PAGE and activity staining with a molecular weight of 99 kDa.

## 1. Introduction

Eukaryotic as well as prokaryotic organisms can produce enzymes adapted to cold. The majority of the cold-adapted enzymes that have been characterized were originated from bacteria living in the Polar Regions in Antarctic and Antarctic seawaters [1, 2]. Other possible sources of cold enzymes are offered by the psychrophiles that inhabit at ~5°C in other permanently cold environments such as the deep sea, glaciers and mountain regions, in soils, and fresh or saline waters related with cold-blooded animals such as fish or crustaceans and artificial sources such as refrigeration appliances and equipments [2–6]. Although the studies of cold adapted enzymes are focusing more on psychrophilic and psychrotrophic bacteria; yeast, fungi, and unicellular green algae that lived in cold environment are also listed as impending sources of cold enzymes. The dominant taxa are ascomycetous and basidiomycetous yeasts and melanized fungi [2, 7].

The properties that characterized and distinguished cold adapted enzymes from enzymes of higher temperature origin are their increased turnover number (*k*_cat_) and inherent catalytic efficiency (*k*_cat_/*K*_*m*_) at low temperatures, which is considered to be an adaptive strategy of the psychrophiles to compensate for low metabolic fluxes and slow reaction rates at their physiological temperatures [8]. Cold-active enzymes are more thermolabile and sensitive to other denaturants relative to their thermophilic and mesophilic counterparts that confer the conformational flexibility to the active site [9]. As temperature decreases, enzymes demonstrate a declining catalytic rate owing to reduction of structural flexibility and undergo cold denaturation [7].

Psychrophilic enzymes possess remarkable prospective in a wide variety of industrial applications. Our interest is focused on cold-adapted protease as a useful biocatalyst in industry such as low temperature clothes washing and enzyme-added detergents to hydrolyze macromolecular stains [10]. An example of commercially available cold-active protease is from Novozyme (trade name Savinase) which is sold as an encapsulated detergent [11]. Proteases are degradative enzymes which catalyze the hydrolysis of proteins and are commonly found among animal tissues, plant, and microorganisms [12]. Proteases execute a large variety of functions and take part in numerous biochemical reactions in living organisms, including formation of spore and germination, coagulation, cascade reactions, posttranslation reactions, modulation of gene expression, enzyme modification, and secretion of various protein enzymes biocatalyst. Over one-third of all known proteolytic enzymes are serine peptidases which constitute the largest group of peptidases [13].

*Glaciozyma antarctica* (formerly known as* Leucosporidium antarcticum*) is an obligate pyschrophilic yeast that inhabit cold, marin, and terrestrial Antarctic ecosystems.* Glaciozyma antarctica* strain PI12 has an optimum growth temperature of 12°C [14] and can grow up to 18°C. The reclassification of this yeast from* L. antarcticum* to* G. antarctica* was proposed by Turchetti et al., 2011  [15]. Previous study on this unique yeast has isolated several cold-active proteins, namely, antifreeze protein, *α*-amylase, and chitinase  [16–18]. In this work, we described the morphological characteristics of* G. antarctica* through observation under scanning electron microscope (SEM) and transmission electron microscope (TEM), cloning of genomic DNA and cDNA sequences encoding the PI12 protease gene, phylogenetic study, and, expression and optimization of the recombinant PI12 protease expression in* Pichia pastoris* expression system.

## 2. Materials and Methods

### 2.1. Culture and Isolation of Microorganism

*Glaciozyma antarctica* strain PI12 was isolated from the Antarctic marine water near Casey Station (66°21′25′′S; 110°37′09′′E). The stock culture was kept in 20% (v/v) glycerol and stored at −80°C prior to experimentation.

### 2.2. Microorganism Identification

The isolated strain was grown on different types of solid media (nutrient agar, sabouroud dextrose agar, and potato dextrose agar) for 10 days at 4°C and the culture characteristics (colony colour, shape, and texture) were determined. Simple and negative staining were performed to identify the cell morphology, arrangement, and size of the psychrophilic yeast. SEM and TEM were conducted to study the surface features and the internal ultrastructure in the thin sections of the* G. antarctica* PI12 cells. Ribosomal RNA identification was performed through ITS1/ITS2 region amplification. The sequence has been deposited in the GenBank database under accession number JX896956 [14].

### 2.3. Nucleic Acid Isolation

A single colony of the yeast was inoculated into 50 mL yeast peptone dextrose broth (YPD) and incubated for 10 days at 4°C without shaking. The cell pellets were frozen in liquid nitrogen and ground to a powder in a ceramic mortar. Genomic DNA was extracted using a phenol-chloroform method as described elsewhere [19]. Removal of RNA from genomic DNA was performed by the addition of 15 *μ*L RNase (10 mg/mL) and incubated at 37°C for 45 min. Total RNA was extracted by the guanidium isothiocyanate method using Trizol reagent (Invitrogen, USA) according to the manufacturer's instructions. Approximately 4 *μ*g of total RNA was digested with 0.5 *μ*g of RNAse-free DNase in a 10 *μ*L reaction mixture containing 1x reaction buffer (20 mM Tris-HCl, pH 8.4; 50 mM KCl and 2 mM MgCl_2_) prior to removal of DNA. The mixture was incubated at 37°C for 15 min. DNaseI was later heat-activated at 65°C for 15 min. DNA and RNA were quantified using Ultraspec 2100 prospectrophotometer (Amersham Biosciences, USA). The integrity of total RNA was assessed by running an aliquot of the RNA sample on a denaturing agarose gel stained with ethidium bromide (EtBr) and by spectrophotometric analysis (Ultraspec 2100 pro Amersham Biosciences, USA). Total RNA was reverse-transcribed with an oligo(dT)_20_ and SuperScript III Moloney Murine Leukemia Virus Reverse Transcriptase (Invitrogen, USA) following the manufacturer's instructions.

### 2.4. Cloning of* G. antarctica *PI12 Protease Gene

Putative partial protease gene of 1321 bp was derived through a genome survey sequence library of* G. antarctica*. The gene has 27% similarity to subtilisin-like protease from* Rhodosporidium toruloides* (EMS20811). DNA walking of partial putative protease gene was conducted using DNA Walking SpeedUp Premix Kit (Seegene, Korea) according to the manufacturer's instructions. Three target specific primers (TSP) were designed from the upstream region of known sequences with the following conditions: 18–23 nucleotides long with 40% < GC content < 60% for TSP1 (5′-AGGGTCAAGACGTTGCAGT-3′), 55°C ≤ Tm ≤ 60°C for TSP2 (5′-ATGCGAAGTCAGAAGCAGGATC-3′) while 60°C ≤ Tm ≤ 65°C for TSP3 (5′-GCAGCCAAATACCTGGAAGCAC-3′). RACE was performed using the SMART RACE cDNA Amplification Kit (Clontech, USA). Two gene-specific primers (GSPs) were synthesized for the 5′- and 3′-RACE reactions based on the sequence of the RT-PCR products as follows: GSP I: 5′-ACCAGTGTCCAGCACCCCAATCTTAATCC-3′ and GSP II: 5′-TCATCAGTGGGACGAGCATGTCGT-3′. Both primers were paired with universal primers provided in the kit to amplify the upstream and downstream region of the gene of interest. The PCR products were subcloned into pGEM-T vector (Promega, USA) and sequenced. Rapid amplification of cDNA ends (RACE) produced two PCR products (5′- RACE and 3′-RACE) which were cloned and sequenced to capture the end-to-end sequence of both products. One set of primers was designed from the extreme 5′ and 3′ ends of cDNA as follows: forward 5′ CAP: 5-GCGGGGGCCGACAATAAAAAC-3 and reverse 3′ A-Tail: 5-TTTTTTTTTTTTTTTTTTTTTTTTTTGAGGTGGC-3′; the 5′-RACE-Ready cDNA served as a template to generate full-length cDNA through long distance PCR (LDPCR). Amplification process was carried out in a reaction mixture (50 *μ*L) containing 5 *μ*L of 50–100 ng cDNA template, 1 *μ*L (10 pmole/*μ*L) of each forward and reverse primers, 1 *μ*L of 50x dNTP mix, 1 *μ*L of 1 U/*μ*L 50x Advantage 2 polymerase mix, 5 *μ*L of 10x Advantage 2 PCR buffer (Clontech, USA), and 36 *μ*L of distilled water. Predenaturation was performed at 95°C for 1 min followed by 30 PCR cycles of denaturation (95°C, 30 sec), annealing (61°C, 3 min), and extension (68°C, 3 min). Final extension was conducted at 68°C for 3 min and held at 4°C. The gene was amplified using thermocycler (CG1-96 Corbett Research, Australia). The amplification of putative open reading frame (ORF) was conducted using both genomic DNA and cDNA to locate the introns and exons locations. The amplicons were examined by electrophoresis, cloned into pJET1.2 blunt vector (Fermentas, USA), and sent for sequencing. The sequence obtained was deposited in GenBank under accession number FM178559.

### 2.5. Assembly and Analysis of PI12 Protease Sequences

Sequences derived from DNA walking and RACE strategy were assembled by conducting sequence alignment in the Biology Workbench (http://workbench.sdsc.edu/). Analysis and interpretation of nucleic acid sequences were conducted using several publicly available web servers and integrated software as listed in Table 1. The phylogenetic tree was constructed based on a comparison of the PI12 protease protein sequence with the closest protease sequence that was extracted from GeneBank database (http://www.ncbi.nlm.nih.gov). The protein sequences used for phylogenetic analysis were aligned using ClustalW. Phylogenetic tree was constructed using Molecular Evolutionary Genetics Analysis 6 (MEGA 6) package by neighbor-joining method.

### 2.6. Cloning of PI12 Protease Gene in* Pichia pastoris* Expression System

The gene encoding mature PI12 protease was amplified by PCR using recombinant pJET1.2/FLcDNA of PI12 protease as a template. Sense primer, 5′-CAAGCCCTAGGCTACGCACGGAACGAGAA-3′, and antisense primer, 5′-CTGCCGAATTCTTCCTCAACCCAGTTACCAAC-3′, were designed based on mature PI12 protease gene sequence (FM178559) for flanking the PCR product at the 5′- and 3′- terminus, respectively. Amplification process was carried out in a reaction mixture (50 *μ*L) containing 5 *μ*L of 50–100 ng DNA template, 1.5 *μ*L (10 pmole/*μ*L) of each sense and antisense primer, 1 *μ*L of 10 mM dNTP mix, 4 *μ*L of 25 mM MgCl_2_, 1 *μ*L of 1 U/*μ*L* Taq* DNA polymerase, 5 *μ*L of 10x PCR buffer (MBI, Fermentas, USA), and 31 *μ*L of dH_2_O. PCR was commenced by predenaturation step at 94°C for 4 min followed by 30 PCR cycles of denaturation (94°C, 1 min), annealing (65°C, 1 min), and extension (72°C, 2 min). Final extension was conducted at 72°C for 7 min and held at 4°C.

The PCR product of the mature PI12 protease gene and the vector pPIC9, (0.5–1 *μ*g) were separately added to restriction enzyme digestion mixtures containing 3 *μ*L* Eco*RI (10 U/*μ*L), 6 *μ*L* Avr*II (10 U/*μ*L), 6 *μ*L Y^+^ Tango buffer (10x), and dH_2_O to a final volume of 30 *μ*L. The reactions were incubated at 37°C for 1 hour. The digestion products were observed through agarose gel electrophoresis and gel purified using the QIAquick Gel Extraction Kit (Qiagen, Germany). The construct was added to a ligation mixture with a ratio of 1 : 5 to pPIC9 vector. The ligation mixture was comprised of 1 *μ*L of 10x ligation buffer (MBI Fermentas, USA), 1 *μ*L T4 DNA ligase (5 U/*μ*L; MBI Fermentas, USA), and dH_2_O to a final volume of 10 *μ*L. The reaction was incubated overnight (14–16 h) at 16°C. Ligation mixture was transformed into* E coli* Top10 competent cells. The procedures for preparation of competent cells and heat-shock transformation were performed according to the method described by Sambrook et al., 1989 [19]. The transformation mixture was spread on LB agar plates containing 100 *μ*g/mL ampicillin and incubated overnight at 37°C. The positive transformants were selected for transformation into* Pichia pastoris* electrocompetent cell.

Prior to transformation into* P. pastoris* cell, the empty pPIC9 and pPIC9 recombinant plasmids harbouring PI12 protease gene were linearized using* Pme*I (10 U/*μ*L) restriction enzyme digestion. The digestion mixtures consisted of 5 *μ*L buffer B (10x), 3 *μ*L of* Pme*I, and 6 *μ*L plasmids while dH_2_O was added to a final volume of 60 *μ*L. The linearized plasmids were purified using the QIAquick PCR purification kit. The preparation of electrocompetent cells was done according to* Pichia* expression kit manual (Invitrogen, USA). Later, about 80–100 *μ*L of the electrocompetent cells were transferred into an overnight prechilled (kept in −20°C) 0.2 cm electroporation cuvette (BioRad, USA) and mixed with 10 *μ*L (1–5 *μ*g) recombinant DNA. The transformation mixtures (mixture of cells and plasmids) were incubated on ice for 5 min in the electroporation cuvette and pulsed with an electroporator (Gene Pulser, BioRad, USA) at the following parameters: charging voltage of 1500 V, capacitance of 25 *μ*F, and resistance of 400 Ω, which generated a pulse length of ~5–10 ms with a field strength of ~7500 V/cm. Iced-cold 1 M sorbitol (1 mL) was added immediately after the pulse into the cuvettes. The contents were transferred into sterile bijou bottles and incubated at 30°C for 2 hours, without shaking. Later, 10–200 *μ*L of the cells were spread on the MD agar [Minimal dextrose medium; 1.34% (w/v) yeast nitrogen base (YNB), 4 × 10^−5%^ (w/v) biotin, 2% (w/v) dextrose, 2% (w/v) agar]. The plates were incubated at 30°C for 3–5 days until the transformant colonies were formed.

### 2.7. Expression and Optimization of Recombinant pPIC9/Mature PI12 Protease

Single colonies of the recombinant* Pichia pastoris* carrying the pPIC9/PI12 protease were grown in 3 mL of BMGY [buffered glycerol-complex medium; 1% (w/v yeast extract), 2% (w/v) peptone, 1.34% (w/v) yeast nitrogen base (YNB), 4 × 10^−5%^ (w/v) biotin, 1% (w/v) glycerol, 100 mM potassium phosphate buffer, pH 6.0], at 30°C in a shake incubator (250 rpm) overnight. The next day, 1 mL of the starter cultures was used as inoculum and inoculated into 10 mL of BMGY in a 50 mL flask and incubated in a condition as described to generate cell biomass before induction. Cells were harvested by centrifugation at 1500 ×g for 10 min.

The cells were resuspended in 50 mL BMMY medium [buffered methanol-complex medium; 1% (w/v) yeast extract, 2% (w/v) peptone, 1.34% (w/v) yeast nitrogen base (YNB), 4 × 10^−5%^ (w/v) biotin, methanol, 100 mM potassium phosphate buffer, pH 6.0] and incubated with vigorous shaking at 30°C to induce expression. The expression control was done by applying the same protein expression condition to the recombinant* Pichia* bearing the empty vector (pPIC9). The growth of* Pichia pastoris* harboring recombinant plasmid GpPro2 was optimized in four different parameters in order to enhance the protease enzyme yield. The yeast was grown up to 5 days for postinduction study and the concentration of methanol was varied from 0% to 1%. Assay temperature of active enzyme was optimized ranging from 10°C until 35°C with 5°C intervals. The last parameter was to find out the capability of cell density in protease production at A_600 nm_ from 1 to 10.

### 2.8. Protease Assay

The proteolytic activity was measured by the modified method of Brock et al., 1982 [20] using azocasein as substrate. Azocasein (0.5%) was dissolved in 0.1 M Tris-HCl-2 mM CaCl_2_, pH 7. The reaction was initiated by the addition of 100 *μ*L of enzyme solution for 30 min at 20°C. An equal volume of 10% (w/v) trichloroacetic acid (TCA) was added to terminate the reaction. The absorbance was read at 450 nm using UV/Visible spectrophotometer Ultraspec 2100 pro (Amersham Biosciences, USA). One unit of azocaseinase activity was defined as the rate of absorbance change of 0.001 per min at 20°C under the standard assay condition.

### 2.9. SDS-PAGE

Sodium dodecyl sulphate polyacrylamide gel electrophoresis (SDS-PAGE) was prepared according to the Laemmli [21] method using 12% polyacrylamide gel in the Mini PROTEAN 3 Cell apparatus (BioRad, USA). After electrophoresis, gels were stained with Coomassie Brilliant Blue R-250 (BioRad, USA).

### 2.10. Activity Staining and Cup Plate Assay

Activity staining was performed as described elsewhere [22]. The supernatant from recombinant* Pichia* clones were applied in this method and loaded into SDS-PAGE [12% (w/v)]. After electrophoresis, the gel was immersed in 20% isopropanol to remove SDS and washed with distilled water (2-3 times) and the gel was transferred into 2% skim milk yeast peptone dextrose (YPD) agar plate. Protease activity on Petri dish was also tested by the activity ring staining or cup plate assay using 2% skim milk YPD agar plate adapted from Poza et al., 2001 [23]. The plate was incubated at 20°C for 5-6 hours.

## 3. Results and Discussion

### 3.1. Microorganism and Culture Condition

**Figure 1:**
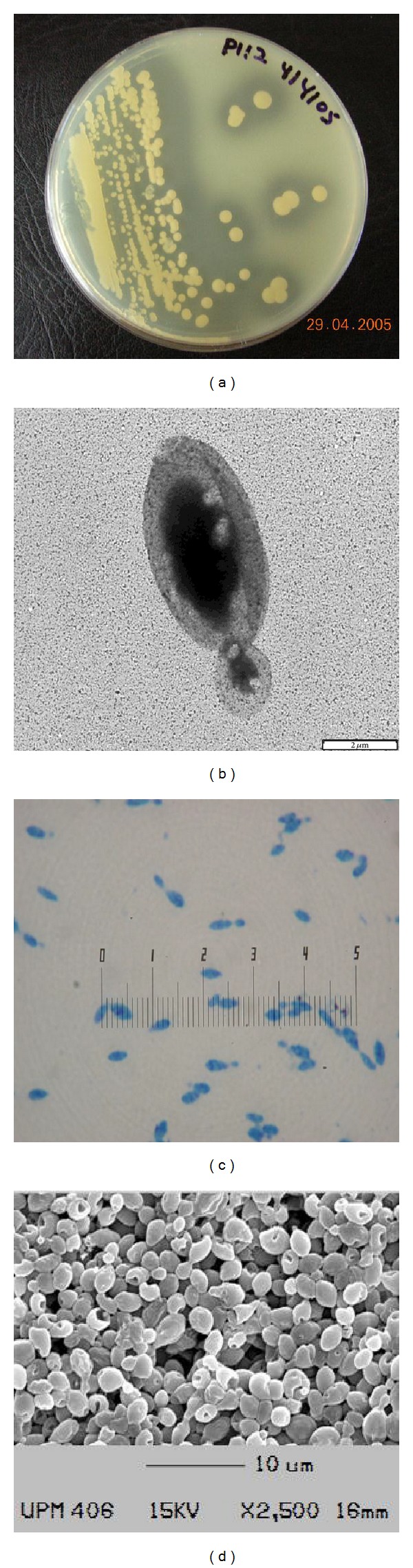
(a) Casein hydrolysis on skim milk agar plate. (b) Negative staining with enlargement of 10,000x, bar = 2 *μ*m. (c) Simple staining photograph. (d) Scanning electron microscopy (SEM) photograph with magnification 2500x. Bar = 10 *μ*m.

After 10 days at 4°C, the growth of* G. antarctica* PI12 on nutrient agar, sabouroud dextrose agar and potato dextrose agar was cream-coloured, smooth, mucous, runny and glistening. The border was entire, circular and convex while on skim milk agar, the yeast produced hydrolytic colonies (Figure 1(a)). The cells of* G. antarctica* PI12 occurred in oval, yeast-like shape with the size of about 2–4 *μ*m and present singly when observed via simple and negative staining (Figures 1(b) and 1(c)). SEM photograph showed budding yeast form of the* G. antarctica* strain PI12 (Figure 1(d)). All cells clumped together and possessed smooth and oval shape appearance.

**Figure 2:**
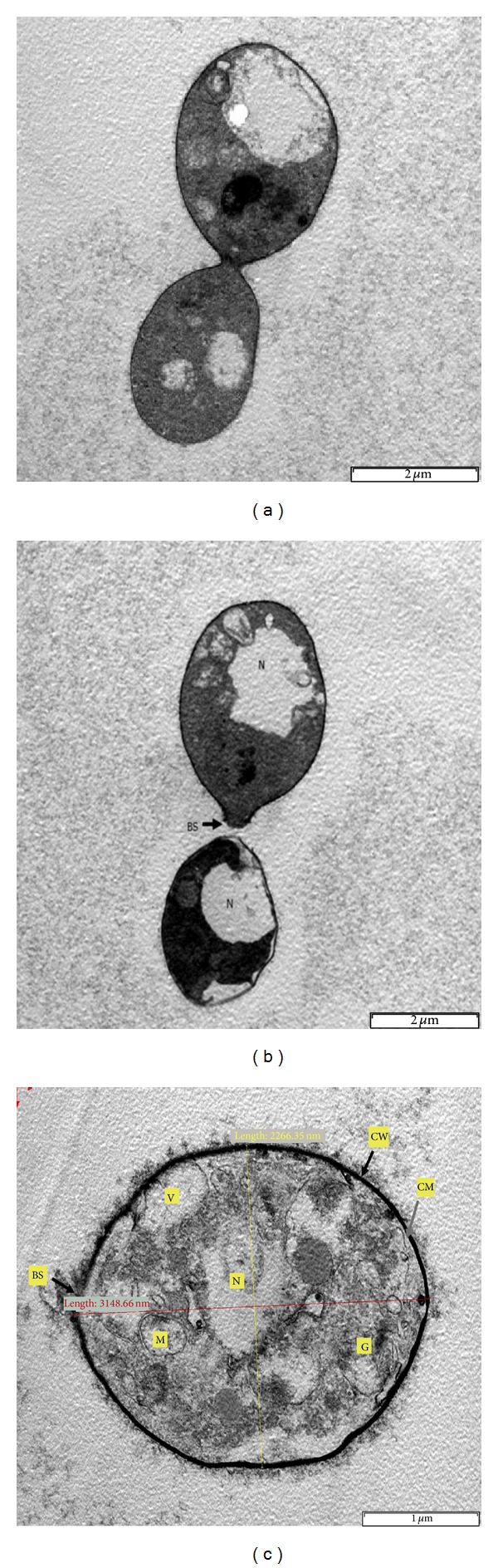
Transmission electron microscopy (TEM) photographs of Antarctic* G. antarctica* strain PI12. (a) 12,500x magnification, (b) 10,000x magnification, (c) 27,500x magnification.The scale bars represent 2 *μ*m for (a) and (b) and 1 *μ*m for (c).

Figure 2 shows TEM photographs of the thin-sectioned of the basidiomycete yeast,* G. antarctica* strain PI12, which reveals the intracellular organelles. Figures 2(a) and 2(b) show budding yeast appearance of membrane-bounded nucleus and easily recognized bud scar while Figure 2(c) illustrates ultrastructure comprising of a dark and thick cell wall, a cell membrane, an irregular shape nucleus, mitochondria, vacuoles, golgi, and bud scars.

### 3.2. Ribosomal RNA Identification

**Figure 3:**
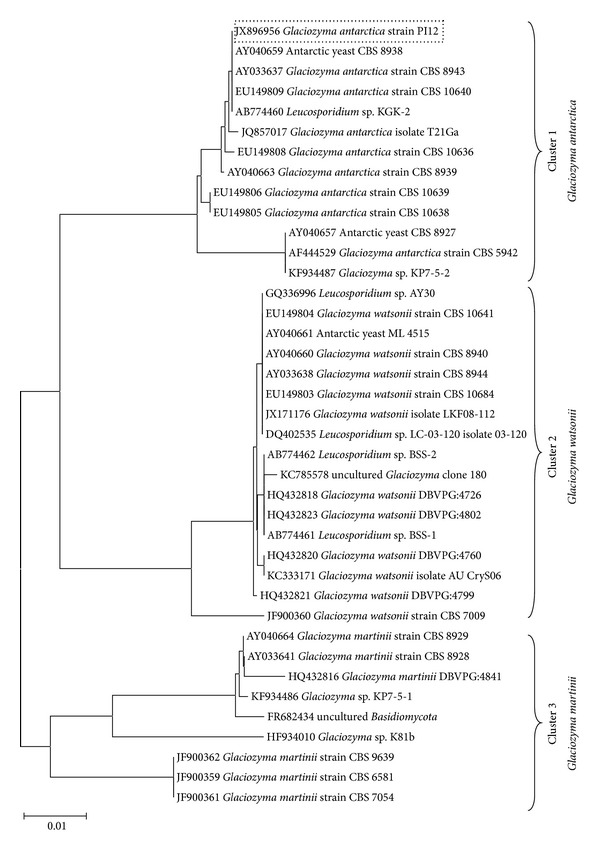
Neighbor-joining phylogenetic analysis of internal transcribed spacer 1 (ITS1)/5.8S rRNA gene/internal transcribed spacer 2 (ITS2) of* Glaciozyma* clade. Numbers indicate percentage bootstrap values calculated on 1000 repeats of the alignment. The tree was constructed using MEGA 6 software [39]. The scale bar represents 0.01 substitutions per nucleotide position. GenBank accession numbers of the sequences are indicated before strain name. ITS1/5.8S/ITS2 sequence of* Glaciozyma antarctica* strain PI12 had been deposited in GenBank with the accession number JX896956 [14].

The ITS1/5.8S rDNA/ITS2 region of PI12 was successfully amplified by Boo et al., 2013 [14] with the predicted size of 646 bp. The ITS1/5.8S/ITS2 sequence of* Glaciozyma antarctica* strain PI12 had been deposited in GenBank with the accession number JX896956. Amplification of ITS region showed 100% identity to ITS1/5.8S/ITS2 sequence of Antarctic yeast CBS 8938 (AY040659) and* Glaciozyma antarctica* strain CBS 8943 (AY033637). National Collection of Yeast Cultures (NCYC) identified PI12 as an isolate of* Glaciozyma antarctica* based on 26S rDNA D1/D2 sequencing result where it displayed 99% sequence identity to* Glaciozyma antarctica* strain CBS 5942 [formerly known as* Leucosporidium antarcticum* strain CBS 5942 (AF444529)]. Phylogenetic tree analysis was created based on comparison of ITS sequence of this strain with the closest strains that are available in the database with reference to BLAST result (Figure 3). Thirty-nine of ITS sequences from* Glaciozyma* clade were compared. The phylogenetic tree constructed consists of three main clusters: cluster I (JX896956* Glaciozyma antarctica* strain PI12, AY040659 Antarctic yeast CBS 8938, AY033637* Glaciozyma antarctica* strain CBS 8943, EU149809* Glaciozyma antarctica* strain CBS 10640, AB774460* Leucosporidium* sp. KGK-2, JQ857017* Glaciozyma antarctica* isolate T21Ga, EU149808* Glaciozyma antarctica* strain CBS 10636, AY040663* Glaciozyma antarctica* strain CBS 8939, EU149806* Glaciozyma antarctica* strain CBS 10639, EU149805* Glaciozyma antarctica* strain CBS 10638, AY040657 Antarctic yeast CBS 8927, AF444529* Glaciozyma antarctica* strain CBS 5942 and KF934487* Glaciozyma* sp. KP7-5-2) is the sequences from strains originally considered as* L. antarcticum*, which was reclassified as* Glaciozyma antarctica* [15]. Cluster II (GQ336996* Leucosporidium *sp. AY30, EU149804* Glaciozyma watsonii* strain CBS 10641, AY040661 Antarctic yeast ML 4515, AY040660* Glaciozyma watsonii* strain CBS 8940, AY033638* Glaciozyma watsonii* strain CBS 8944, AB774462* Leucosporidium* sp. BSS-2, KC785578 Uncultured Glaciozyma clone 180, AB774461* Leucosporidium* sp. BSS-1, EU149803* Glaciozyma watsonii* strain CBS 10684, HQ432823* Glaciozyma watsonii* DBVPG:4802, HQ432818* Glaciozyma watsonii* DBVPG:4726, HQ432820* Glaciozyma watsonii* DBVPG:4760, JX171176* Glaciozyma watsonii* isolate LKF08-112, DQ402535* Leucosporidium* sp. LC-03-120 isolate 03-120, JF900360* Glaciozyma watsonii* strain CBS 7009, KC333171* Glaciozyma watsonii* isolate AU CryS06, HQ432821* Glaciozyma watsonii* DBVPG:4799) is categorized as* G. watsonii* while cluster III (AY040664* Glaciozyma martinii* strain CBS 8929, AY033641* Glaciozyma martinii* strain CBS 8928, HQ432816* Glaciozyma martinii* DBVPG:4841, KF934486* Glaciozyma* sp. KP7-5-1, FR682434 Uncultured Basidiomycota, HF934010* Glaciozyma* sp. K81b, JF900362* Glaciozyma martinii* strain CBS 9639, JF900359* Glaciozyma martinii* strain CBS 6581, JF900361* Glaciozyma martinii* strain CBS 7054) is categorized as* G. martini*. Recognition of the Glaciozyma species has been done by comparing their molecular phylogenetic data and physiological and morphological characteristics [15]. Phylogenetic analysis of ITS1/5.8S/ITS2 sequence of strain PI12 showed that the sequence was closely related to ITS1/5.8S/ITS2 sequence of* Glaciozyma antarctica* with high homology similarity to Cluster I (99-100% identity). The ITS1/5.8S/ITS2 sequence of* G. antarctica* strain PI12 was differed from* G. watsonii* (Cluster II) by 2-3% gap in the sequence (93% identity) and* G. martinii* by 4-5% gap in the sequence (90–92% identity).

### 3.3. Sequence Analysis of PI12 Protease cDNA

In this study, amplification of the PI12 protease gene sequence was conducted using genomic DNA and cDNA as a template. Putative partial PI12 protease gene with the size of 1321 bp was initially acquired from the* G. antarctica* genome survey sequence library (Supplementary Figure; see Supplementary Material available online at http://dx.doi.org/10.1155/2014/197938). However, the gene was discovered as an incomplete protease gene sequence. DNA walking strategy successfully amplified 1500 bp of the upstream region of partial PI12 protease. Both sequences of about 1500 bp (derived from the DNA walking reaction) and 1321 bp (partial PI12 protease) were assembled and made up a total length of 2687 bp protease sequence (Supplementary figure). The putative PI12 protease was predicted by Augustus webserver (http://augustus.gobics.de/) having intervening sequences; introns. Thus, reverse-transcriptase PCR (RT-PCR) and rapid amplification of cDNA ends (RACE) were accomplished to obtain the full-length cDNA. As a result, the ORF comprising 2892 bp encoding 963 amino acid residues were successfully deduced.

**Figure 4:**
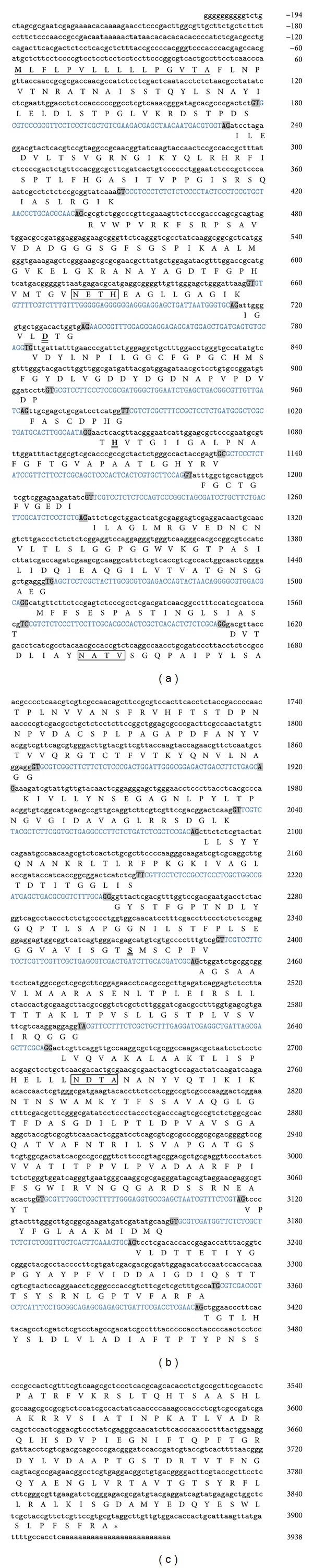
Nucleotide and deduced amino acid sequences of genomic DNA and cDNA encoding protease from* G. antarctica* strain PI12. The nucleotides are numbered from the 5′ end of the cDNA. A putative CAAT, TATA box, and polyadenylation signal are bold italic. The putative N-terminal signal peptide is underlined. The initiation codon is boldfaced, and termination codon is represented by an asterisk (∗), boxed peptide sequences showed N-glycosylated asparagines residues. The amino acid residues that form the catalytic triad are double underlined and bold while introns are represented in blue font. Highlighted in grey are the intron donor-acceptor splice sites.

Nucleotide and deduced amino acid sequences of genomic DNA and cDNA fragment encoding PI12 protease were shown in Figure 4. The size of the ORF of putative PI12 protease was 3867?bp with genomic DNA as the template while the ORF comprised 2892?bp encoding 963 amino acid residues were successfully deduced through RACE strategy with cDNA as the template. The ORF was predicted using ORF Finder (http://www.ncbi.nlm.nih.gov/projects/gorf/) while prediction using SignalP (http://www.cbs.dtu.dk/services/SignalP/) webserver allowed the identification of the putative PI12 protease prepeptide where it predicted the presence of signal peptidase I cleavage sites [24]. The putative N-terminal signal peptide (1 to 16; the underlined amino acid) was identified as a secretion signal. There is no propeptide predicted by ProP Webserver (http://www.cbs.dtu.dk/services/ProP-1.0/). Comparison of cDNA and gene sequences allowed determination of gene structure. Therefore, sequence alignment was performed between the sequence of these two templates and it was confirmed that the coding sequence (consisted of 19 exons) was interrupted by 18 introns with their length varying from 48 to 60 nucleotides. However, the GT and AG rule for donor-acceptor sites was not obeyed. Of 18 introns, 10 introns contained canonical GT and AG dinucleotides at their 5′ and 3′ ends while 8 introns hold noncanonical splice site pairs with TT-GG (2 introns), AG-TG, GC-GT, TG-GG, TC-GG, TA-GG, and TG-AG dinucleotides at the splice junction. Upper case letters in blue colour represent introns while lower case letters in black colour represent exons. Splice donor and acceptor were highlighted in grey.

The amino acid composition (upper case letters) within the ORF amino acid residues was determined by the ProtParam tool of the Expasy molecular biology server (http://www.expasy.org/tool). Translation starts at a nucleotide position 1 and translation stop is marked with an asterisk. The nucleotide sequences ATG (1 to 3) and TAG (3865 to 3867) indicate the initiation codon and terminal codon, respectively. The presumed putative sequences of promoter CAAT (−101 to −98) and TATA-box (−92 to −88) were identified in the 5′-untranslated (UTR) region. The search was achieved via Neural Network Promoter Prediction (Berkeley Drosophila Genome Project) (http://www.fruitfly.org/seq_tools/promoter.html). In the 3′-untranslated (UTR) region, there was a putative polyadenylation signal,* attaa* (3889 to 3893) detected from the TAG stop codon to the poly (A) tail. The predicted molecular mass and pI of PI12 serine protease were 100.99 kDa and 6.41, respectively. Out of 963 amino acid residues in the deduced sequence of the PI12 protease, 70 are negatively charged residues (Asp + Glu), while 66 are positively charged residues (Arg + Lys) at neutral pH (Table 2). The total charged residues (Arg, Asp, Glu, His, and Lys) are 150 amino acids in the open reading frame of PI12 protease corresponded to 15.6%, while hydrophobic residues (Ala, Phe, Ile, Leu, Met, Pro, Val, and Trp) are 441 amino acids which corresponded to 45.8% of the total amino acids discovered. The total uncharged residues (Asp, Cys, Gln, Gly, Ser, Thr, and Tyr) are consisted of 372 amino acids, which corresponded to 38.6% of the total amino acids of the open reading frame of PI12 protease. The aliphatic index is 92.90 and the grand average of hydropathicity (GRAVY) is 0.141. The instability index (II) of PI12 protease was computed to be 34.14 which classify the protein as stable.

N-glycosylation sites were predicted using NetNGlyc 1.0 Server (http://www.cbs.dtu.dk/services/NetNGlyc). There were five putative N-glycosylation sites for the PI12 protease gene predicted using the server. For N-glycosylation, the sequence motif Asn-Xaa-Ser/Thr (Xaa any amino acid, but not Pro) has been defined as a prerequisite for glycosylation. The sequence motifs in PI12 protease occurred in the position 170 NETH, 365 NATV, 576 NLTP, 634 NDTA, and 833 NSSP. Among five predicted sites, only three (except positions 576 and 833) showed a high potential score (averaged output of nine neural networks) which support the prediction. The putative N-glycosylation sites were demonstrated in boxes in Figure 4. The comparison of amino acid composition of this cold-adapted protease from* G. antarctica *strain PI12 with nine other subtilisin serine proteases from different families is shown in Table 2. Analysis of this comparison depicts that the percentage of each amino acids in PI12 protease is almost the same with other proteases while Gly (G) is the most abundant, accounting for up to 10.4% of the total amino acid in PI12 protease. PI12 protease also contained the highest percentage of Leu (L) with 9.9% of the total amino acids compared to other proteases. Hydrophobic profile of PI12 amino acid protease was performed by ProtScale tools with Kyte and Doolittle method at http://au.expasy.org/cgi-bin. The hydrophobicity profile elucidation of PI12 protease amino acid was carried out from amino acid 1 to 963. The amino acid residues from 1 to 16 (M-L-F-L-P-V-L-L-L-L-L-P-G-V-T-A) and 548 to 562 (V-A-V-I-S) were hydrophobic grooves. The first region which consisted of 16 amino acids was very hydrophobic and was predicted to be the signal peptide by SignalP and ProP webserver.

**Figure 5:**
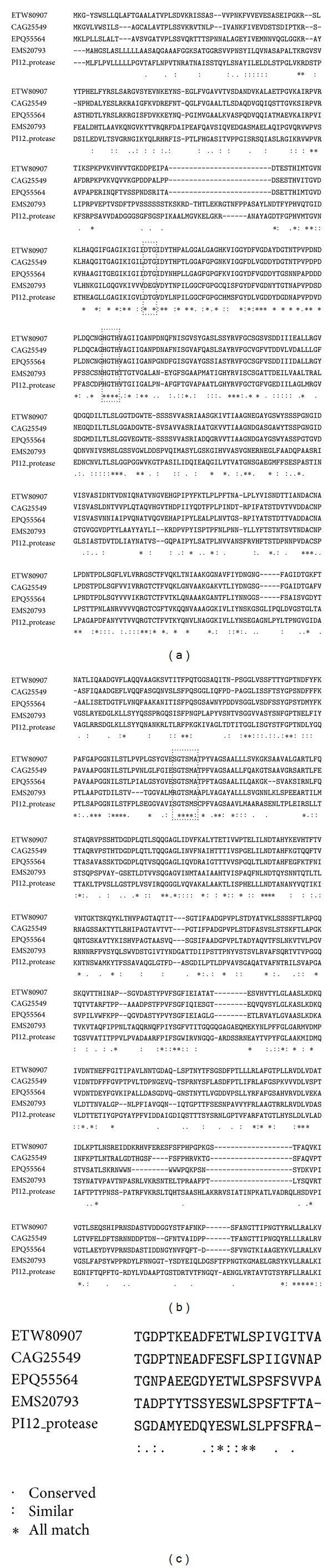
Multiple sequence alignment between amino acid sequence of PI12 protease with other subtilisin. The serine protease sequences were obtained from the GenBank database. Their accession numbers are: EMS20793 (subtilisin-like protease of* Rhodosporidium toruloides* NP11), CAG25549 (subtilisin-like protease of* Pleurotus ostreatus*), EPQ55564 (subtilisin-like protease of* Gloeophyllum trabeum* ATCC 11539), and ETW80907 (serine protease S8 of* Heterobasidion irregulare* TC 32-1). Putative ORF of serine protease from* G. antarctica* PI12 was labeled as PI12 protease.

The bold and double-underlined amino acids showed residues of catalytic triad, aspartic acid ($\underline{\underline{D}}$), histidine ($\underline{\underline{H}}$), and serine ($\underline{\underline{S}}$) active site, respectively. NCBI Conserved Domains Database (CDD) identified the presence of subtilase, protease and subtilisin-like serine protease domains in the amino acid sequence. The predicted PI12 protease amino acid sequence was found sharing significant homology to the subtilisin family of protease. The highest similarity of PI12 protease was observed with 42% identity to subtilisin-like protease of* Rhodosporidium toruloides* (EMS20793), 37% identity to subtilisin-like protease of* Pleurotus ostreatus* (CAG25549), and 35% identity to subtilisin-like protease of* Gloeophyllum trabeum* ATCC 11539 (EPQ55564). The nucleotide sequence of* G. antarctica *strain PI12 showed low homology to the fungus subtilisin protease family and none to other psychrophilic proteases. The multiple sequence alignment was illustrated in Figure 5. The consensus sequence around the catalytic triad, aspartic acid (D), histidine (H), and serine (S) of PI12 protease showed high conservation with other subtilisin (showed in black boxes). Glycine residues G^142^, G^173^, G^191^, G^262^, and G^327^ that were highly conserved in most of subtilases were conserved in PI12 protease [25]. Therefore, PI12 protease belongs to the subtilisin subgroup of the subtilase serine protease superfamily. This serine protease of* G. antarctica *PI12 has been deposited into GenBank with accession number CAQ76821.

### 3.4. Phylogenetic Relationship of PI12 Protease

**Figure 6:**
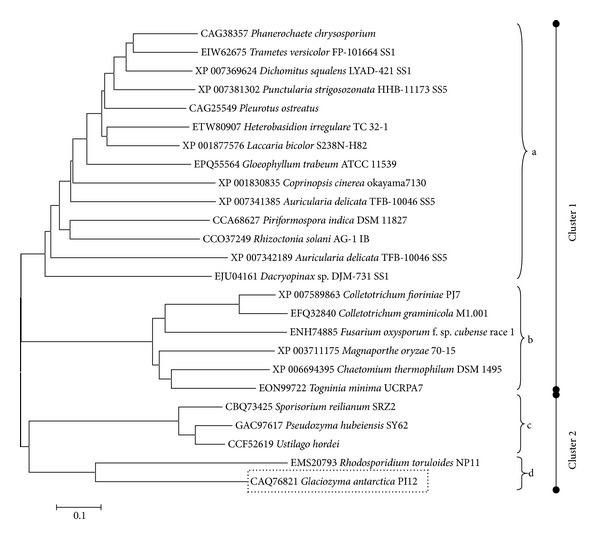
Phylogenetic dendrogram among 25 translated amino acid sequences of serine protease. The tree was constructed using a neighbor-joining algorithm with 1000 bootstrappings by MEGA 6 software [39]. The scale bar represents 0.1 substitutions per amino acid position. The serine protease sequences were obtained from the GenBank database. GenBank accession numbers of the sequences were indicated before the strain name. The putative serine protease of* G. antarctica* strain PI12 has been deposited in GenBank with accession number CAQ76821.

The phylogenetic tree was constructed to demonstrate the similarities and relationship of protein sequence between different species or organisms.  Figure 6 illustrates the phylogenetic dendrogram indicating the relationship of PI12 protease with another 25 translated amino acid sequences of serine protease. The bar scale signifies the branch length to approximate variance along each branch. Basically, this tree comprises two main clusters: cluster I was subdivided into subcluster a (CAG25549, EPQ55564, ETW80907, XP_007381302, XP_001877576, CAG38357, XP_007341385, CCA68627, EJU04161, XP_007369624, EIW62675, CCO37249, XP_001830835, XP_007342189) and subcluster b (XP_007589863, XP_003711175, EFQ32840, XP_006694395, ENH74885, EON99722); cluster II was subdivided into subcluster c (CBQ73425, GAC97617, CCF52619) and subcluster d [CAQ76821 (PI12 protease), EMS20793].

Serine protease constructed by the phylogenetic tree comprised large size of protein with about 801 to 1032 amino acids. The majority of the translated protease sequences were obtained from fungus. All serine proteases from cluster 1 subcluster a are derived from* Agaricomycetes* except for EJU04161, which belongs to* Dacrymycetes* whereby all serine proteases from cluster 1 subcluster b are from* Sordariomycetes*. Cluster II subcluster c consisted of serine protease from fungus belonging to* Ustilaginomycetes*. PI12 protease was categorized under cluster II subcluster d. It possessed homology similarity with the subtilisin-like protease from* Rhodosporidium toruloides* (EMS20793) (formerly known as* Rhodotorula glutinis* or* Rhodotorula gracilis*), which is a pink-coloured nonpathogenic oleaginous yeast (42% identity). Both* G. antarctica* PI12 and* Rhodosporidium toruloides *NP11 belong to Microbotryomycetes class. PI12 protease did not show any homology similarity to other psychrophilic proteases; however, it showed 37% identity to hypothetical protein from* Pseudozyma antarctica *T-34 which was isolated from Japan (GAC76005) (not listed in the phylogenetic tree).

### 3.5. Cloning and Expression of Cold-Adapted PI12 Protease in the Yeast Expression System

The PCR product harbouring the mature PI12 protease with two flanking restriction sites (*Eco*RI and* Avr*II) was cloned into pPIC9 expression vector. The recombinant plasmid harbouring PI12 protease was used to transform both GS115 and KM71 intended to produce both His^+^ Mut^+^ and His^+^ Mut^S^. Two recombinant clones from GS115 (His^+^ Mut^+^) strain (GpPro1 and GpPro2) and one from KM71 (His^+^ Mut^S^) strain (KpPro1) were successfully obtained which were further analyzed for protein expression study.

### 3.6. Expression and Enzyme Assay of PI12 Protease in* Pichia pastoris*

All positive recombinants were grown in small scale cultures and assayed for the cold-adapted protease activity in the culture supernatants. The clones were first grown in BMGY medium with glycerol as the sole carbon source. For induction of* AOX1* promoter and protein production, the growth medium was transferred to the same medium except with 0.5% methanol in place with glycerol as the carbon source [26]. The supernatant of the recombinant* P. pastoris* expression cultures after 48 h of incubation time was analyzed to determine the recombinant protease activity by the modified method of Brock et al. (1982) [20]. All tested clones secreted protease into the supernatant in different expression levels. This might be due to the variability of the strength of the promoter and copy number in each cell to secrete and express the recombinant product. The clone with the highest yield was GpPro2 with 6.3 U/mL activity followed by KpPro1 and GpPro1 with 2.5 U/mL and 3.7 U/mL activities, respectively (data not shown). It is perceptible that expression system of* Pichia pastoris* is the ideal system for this protein. The preliminary assay has fruitfully produced high protease expression at 20°C. Yet, further optimization of the recombinant PI12 protease production need to be conducted in order to examine the optimum condition of this protein. Reported cold-adapted proteases showed optimum activity at a range of 15°C–60°C [27–30]. This difference was possibly related to the respective genetic and physiological adaptation of the strains. There are several factors that influenced the different rate of expression. It includes different site of integration between interest gene and* Pichia* genome, variability of different expression host cell to secrete and express the recombinant enzyme, and different sites in the polylinker for inserting foreign genes [31].

This is the second extracellular protease reported to be synthesized by this obligate psychrophilic yeast,* G. antarctica*. The first cold-adapted extracellular subtilase from this yeast has been purified and characterized previously [32, 33]. The extracellular serine proteinase (LAP2) from this Antarctic yeast, formerly known as* Leucosporidium antarcticum *strain 171, is specific towards synthetic substrates of chymotrypsin and subtilisin. The subtilase of* L. antarcticum *strain 171, showed specific activity of 40.43 U/mg when utilized* N*-SucAAPF*p*NA as a substrate [33].

### 3.7. Optimization of Protease Production

**Figure 7:**
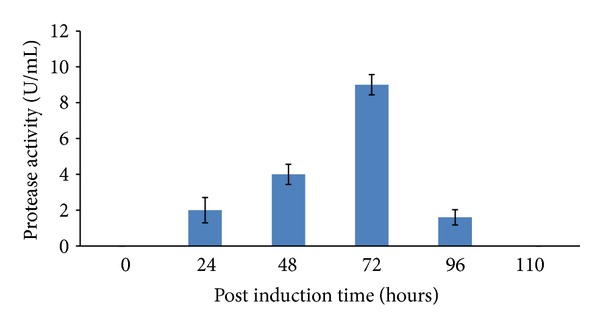
Effect of postinduction time towards protease activity. The highest activity was detected at 72 hours after 110 hours of incubation, with 9 U/mL activity followed by 48, 24 and 96 hours. The substrate (azocasein) was dissolved in Tris HCL buffer and incubated for 30 min at 20°C. The absorbance was measured at 450 nm after 15 min incubation. A one-way ANOVA was used to test for preference differences among six different postinduction times. The statistical value (*P* value <0.05) between hours is 0.003.

GpPro2 was chosen for further optimization due to its highest protease activity at the preliminary expression stage. High protease activity was detected after 72 hours of incubation (Figure 7) by using the standard protocol method as recommended for* Pichia pastoris* expression system (*Pichia* Expression Kit, Invitrogen USA). The expression decreased after 72 hours and no activity was detected at 110 hours. The prolonged incubation may result in the decrease of biomass production and will subsequently reduce the expression of recombinant protein. In addition, oxygen and nutrient depletion during prolonged incubation will trigger the stress responses in order to survive in such harsh environment [34].

**Figure 8:**
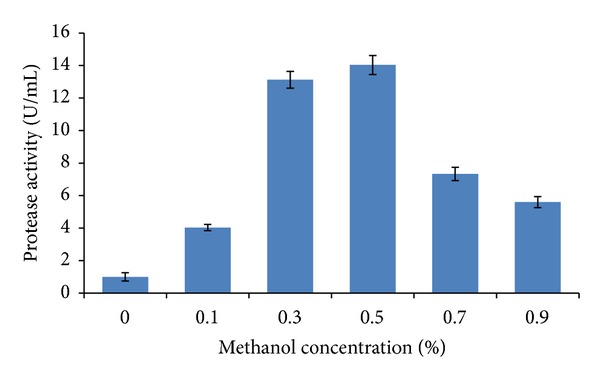
Effect of methanol concentration towards protease activity. The recombinant was cultivated for 72 hours at 30°C in BMMY medium. 0.5% methanol gives the highest activity with 14 U/mL. The substrate (azocasein) was dissolved in Tris HCL buffer and incubated for 30 min at 20°C. The mixture was left for 15 min and absorbance was taken at 450 nm. A one-way ANOVA was used to test for preference differences among six different concentrations. The statistical value (*P* value <0.05) between concentration is 0.02.

Methanol was used as a sole carbon by* P. pastoris*. The methanol was oxidized to formaldehyde with the aid of oxygen as electron acceptor by an alcohol oxidase enzyme (AOX gene) [35]. The optimal methanol concentration is at 0.5% (v/v) with 14 U/mL activity. The addition of more than 0.5% (v/v) methanol resulted in tremendous decrease of protease activity (Figure 8). Production of PI12 protease was decreased when the methanol concentration increased. This might be due to the toxic effect of methanol concentration on DNA replication and membrane synthesis. Basically, methanol is used to increase the permeability of the cell membrane to help the secretion of foreign protein [26]. However, if the permeability of cell membrane is too high, low molecular size metabolites would leak from the cell and such leakage will seriously damage cell activities. Such condition would abuse the cellular system and might reduce the yield of protein expression [36].

**Figure 9:**
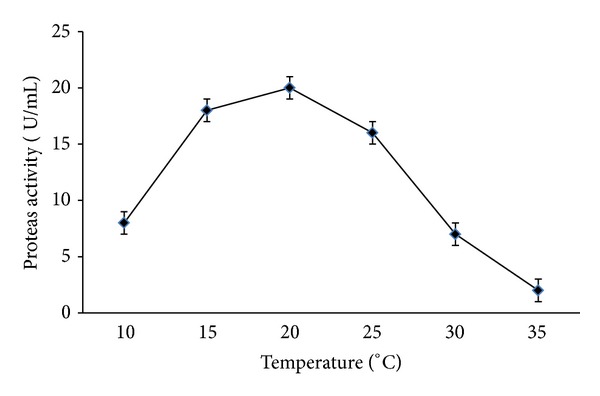
Effect of temperature towards protease activity. The reaction was carried out in temperature ranging from 10°C to 35°C at pH 7 for 30 min. A one-way ANOVA was used to test for preference differences among six different temperatures. The statistical value (*P* value <0.05) between temperature is 0.067.

Psychrophilic microorganism dominated in low temperature habitat which most other microorganism are incapable of withstanding due to their inability to produce enzyme that can tolerate the extreme environment condition. Psychrophilic enzymes are always associated with high catalytic efficiency at low and moderate temperature (0°C to 30°C) compared to the mesophilic counterpart that are generally unable to catalyze efficiently under extremely low temperature [2]. The recombinant protein assay was done at various temperatures ranging from 10°C to 35°C using 0.5% azocasein as a substrate dissolved in 50 mM Tris HCl in presence of 2 mM CaCl_2_, pH 7. In the activity assays, expressed protease showed a highest activity at 20°C (20 U/mL) and decreased tremendously once it exceeded maximum temperature due to the denatured state of the enzyme (Figure 9). The active site of an enzyme is easily altered when the high temperature is introduced during incubation or activity assay, resulting in a low reaction rate [37].

**Figure 10:**
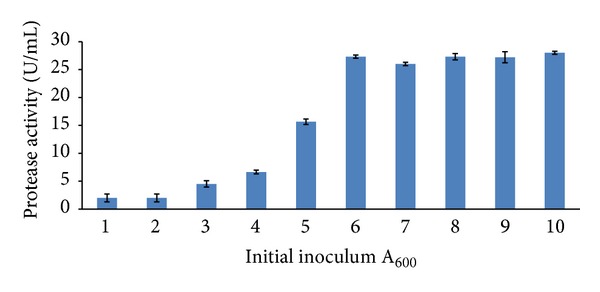
Effect of inoculums size towards protease activity. The recombinant was cultivated for 72 hours at 30°C in BMMY medium with 0.5% methanol concentration. A one-way ANOVA was used to test for preference differences among six different optical densities. The statistical value (*P* value <0.05) between optical density value is 0.03.

The recombinant construct was transferred into BMMY media at different A_600 nm_ ranging from 1 to 10. The highest activity of active enzyme was detected at A_600 nm _6.0 (28.3 U/mL) and above (Figure 10). The slope during exponential phase was much higher compared to other phases indicating an increase in the doubling time. At exponential phase, the cells are adapted to the growth condition and other processes like cell growth cycle and synthesis of RNA. The yield of active enzyme during expression of* P. pastoris* dropped after 28 hours of incubation, which is at stationary or death phase [38].

### 3.8. Detection of Recombinant Protein by SDS-PAGE and Activity Staining

**Figure 11:**
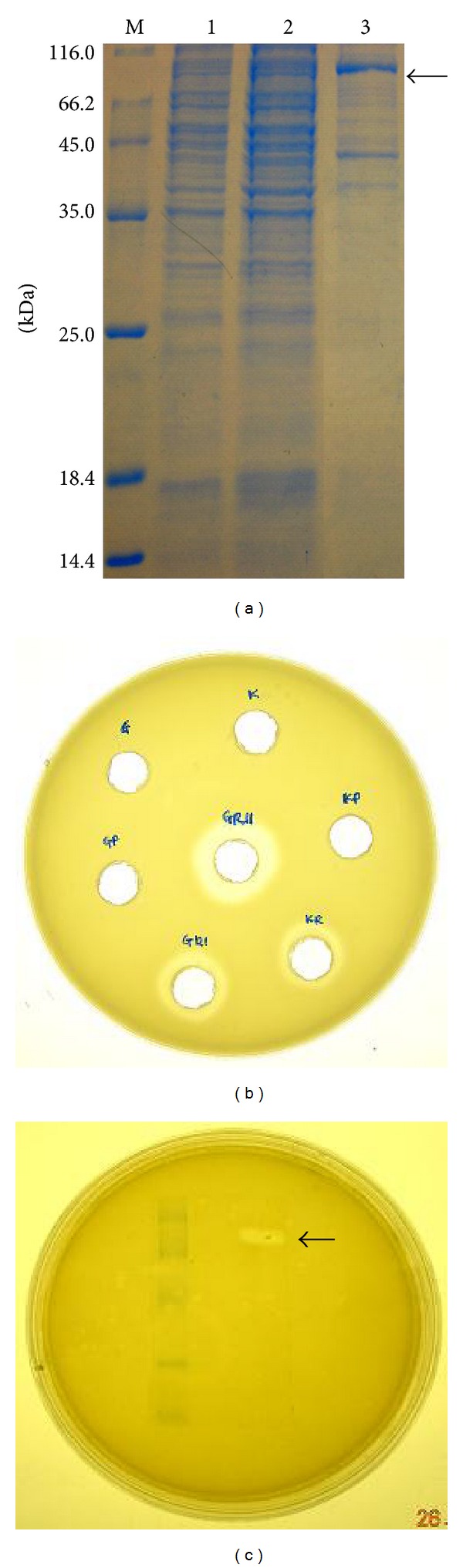
(a) Coomassie-stained SDS-PAGE of recombinant* P. pastoris* clone. 1 mL supernatants collected from the third-day culture were concentrated 10-fold using trichloroacetic acid, and 10 *μ*L resultant products were applied to SDS-PAGE analysis. Lane 1: molecular weight marker (Fermentas); Lane 2: GS115 (Control); Lane 3: GS115/pPIC9 empty (Control); Lane 4: GS115/pPIC9/PI12prot (GpPro2). The arrow indicates the secreted recombinant PI12 protease. (b) Plate activity staining of different recombinant* P. pastoris* clones. G: GS115; GP: GS115/pPIC9; GRI: GS115/pPIC9/PI12prot1 (GpPro1); GRII: GS115/pPIC9/PI12prot2 (GpPro2); K: KM71; KP: KM71/pPIC9; KR: KM71/pPIC9/PI12prot (KpPro). (c) Gel activity staining of recombinant PI12 protease in* P. pastoris* GpPro2 clone. Lane 1: prestained molecular weight marker (Fermentas); Lane 2: GS115/pPIC9 empty (Control); Lane 3: GS115/pPIC9/PI12prot1 (GpPro2). The arrow indicates the secreted recombinant PI12 protease.

The SDS-PAGE gel of the GpPro2 recombinant clones' supernatant showed a putative expression band at around 99.3 kDa. Plate staining and gel activity staining were executed to examine if the recombinant PI12 protease expressed in* P. pastoris* was functionally active. In the plate staining, the transformants showed strong proteolytic activity (Figure 11(a)). In gel staining, proteolytic activity was also detected around 99.3 kDa after incubation at 20°C. This is evidence of protease secretion into the surrounding media whereby it catalyzed the breakdown of casein, thus, formed clearing zones around the activity ring and band (Figures 11(b) and 11(c)). As a conclusion, the cold-adapted PI12 protease was functionally expressed in* P. pastoris* GS115 (GpPro2 clone) with the size of 99.3 kDa.

## 4. Conclusions

Psychrophilic yeast, which was originated from marine water in Casey Station, Antarctica, was identified as* Glaciozyma antarctica* strain PI12. A serine protease with an open reading frame of 2892 bp encoded for 963 amino acids from this Antarctic yeast has been cloned and expressed in* Pichia* expression system. This is the second extracellular protease reported being synthesized by this psychrophilic yeast. Sequence analysis through bioinformatics studies revealed few homology identities of this protein with other serine proteases. The mature PI12 protease was successfully expressed and secreted into the culture medium driven by the* Saccharomyces cerevisiae* *α*-factor signal sequence in* P. pastoris.* The protease production in* P. pastoris* was best obtained from strain GS115 (GpPro2) with 28.3 U/mL after 72 hours of induction time with 0.5% (v/v) of methanol inducer. The starting optical density at 600 nm (OD600) was 6.0 and the optimum assay temperature was 20°C. The expressed protein was detected by SDS-PAGE and activity staining with a molecular weight of 99 kDa. This cold-active enzyme might offer an opportunity for biotechnological exploitation based on its high catalytic activity at low temperatures and low thermostability. Structural and functional study of this cold-adapted enzyme would give insight into its adaptation to an extreme cold environment.

## Supplementary Material

Genomic DNA sequence assembly of PI12 serine protease derived from DNA walking and genome sequence library (4081 bp) (5' to 3').

## Figures and Tables

**Table 1:** List of web servers applied for full-length cDNA of PI12 protease analysis.

Analysis	Web server/Software	Website
(1) Open reading frame (ORF) (2) Amino acid translation (3) Intron-exon boundaries	Augustus Web server	http://augustus.gobics.de/

(1) Amino acid composition (2) Molecular mass (3) Isoelectric point	ProtParam	http://www.expasy.org/tool

Homology search	(1) NCBI BLASTX	http://www.ncbi.nlm.nih.gov
	(2) NCBI BLASTP	

Phylogenetic tree	(1) Molecular Evolutionary Genetics Analysis (MEGA 6)	http://www.megasoftware.net/
	(2) CLUSTALW	http://seqtool.sdsc.edu/CGI/BW.cgi

Propeptide	ProP	http://www.cbs.dtu.dk/services/ProP-1.0/

Open reading frame	ORF Finder	http://www.ncbi.nlm.nih.gov/projects/gorf

Signal peptide	SignalP	http://www.cbs.dtu.dk/services/SignalP

Hydrophobic profile	ProtScale	http://au.expasy.org/cgi-bin

Multiple sequence alignment	Biology Workbench	http://workbench.sdsc.edu/

Protein motifs	ScanProsite	http://prosite.expasy.org/scanprosite/

N-glycosylation	NetNGlyc	http://www.cbs.dtu.dk/services/NetNGlyc/

**Table 2:** Amino acid composition and molecular weight of proteases across different families.

Amino		CAQ76821		CAG25549		EDR11679		CAG38357		CAD11898		CAL25578		EDU50747		AAW40780		ACB30121		ACB30119	
acid		Qty	%	Qty	%	Qty	%	Qty	%	Qty	%	Qty	%	Qty	%	Qty	%	Qty	%	Qty	%
Ala	(A)	97	10.1	81	9.1	78	8.7	75	9.0	82	9.9	101	11.5	81	8.9	91	9.6	80	9.2	105	11.6
Arg	(R)	40	4.2	27	3.0	22	2.4	16	1.9	17	2.1	18	2.1	29	3.2	22	2.3	21	2.4	31	3.4
Asn	(N)	44	4.6	38	4.3	43	4.8	41	4.9	42	5.1	40	4.6	52	5.7	51	5.4	45	5.2	43	4.7
Asp	(D)	49	5.1	62	6.9	54	6.0	65	7.8	55	6.7	51	5.8	53	5.8	66	6.9	52	6.0	63	7.0
Cys	(C)	8	0.8	5	0.6	4	0.4	4	0.5	9	1.1	8	0.9	9	1.0	9	0.9	7	0.8	10	1.1
Gln	(Q)	23	2.4	34	3.8	23	2.6	28	3.4	30	3.6	37	4.2	35	3.9	29	3.1	19	2.2	26	2.9
Glu	(E)	21	*2.2 *	16	1.8	19	2.1	20	2.4	15	1.8	20	2.3	25	2.8	26	2.7	28	3.2	24	2.6
Gly	(G)	100	10.4	89	10.0	84	9.3	85	10.2	85	10.3	77	8.8	84	9.3	80	8.4	85	9.8	86	9.5
His	(H)	14	1.5	14	1.6	13	1.4	16	1.9	13	1.6	11	1.3	16	1.8	14	1.5	16	1.8	12	1.3
Ile	(I)	53	5.5	49	5.5	51	5.7	53	6.4	41	5.0	38	4.3	51	5.6	44	4.6	35	4.0	44	4.9
Leu	(L)	95	9.9	68	7.6	71	7.9	62	7.4	60	7.3	66	7.5	67	7.4	80	8.4	74	8.5	68	7.5
Lys	(K)	26	2.7	29	3.2	44	4.9	29	3.5	49	5.9	51	5.8	40	4.4	30	3.2	50	5.8	56	6.2
Met	(M)	12	1.2	2	0.2	2	0.2	9	1.1	11	1.3	8	0.9	18	2.0	8	0.8	11	1.3	15	1.7
Phe	(F)	41	4.3	59	6.6	47	5.2	55	6.6	29	3.5	36	4.1	44	4.9	39	4.1	34	3.9	34	3.8
Pro	(P)	62	6.4	65	7.3	67	7.4	58	7.0	58	7.0	59	6.7	50	5.5	51	5.4	55	6.4	57	6.3
ser	(S)	77	8.0	69	7.7	91	10.1	60	7.2	66	8.0	70	8.0	72	7.9	111	11.7	63	7.3	61	6.7
Thr	(T)	87	9.0	89	10.0	75	8.3	83	10.0	65	7.9	73	8.3	71	7.8	72	7.6	71	8.2	68	7.5
Trp	(W)	5	0.5	3	0.3	6	0.7	2	0.2	12	1.5	12	1.4	12	1.3	9	0.9	12	1.4	14	1.5
Tyr	(Y)	33	3.4	12	1.3	26	2.9	7	0.8	29	3.5	28	3.2	39	4.3	50	5.3	26	3.0	27	3.0
val	(V)	76	7.9	82	9.2	80	8.9	66	7.9	57	6.9	74	8.4	58	6.4	68	7.2	82	9.5	62	6.8

Total		963	100.0	893	100.0	900	100.0	834	100.0	825	100.0	878	100.0	906	100.0	950	100.0	866	100.0	906	100.0
MW	(Da)	100989.8		93266.9		94436.5		86945.5		86954.9		92490.5		97934.3		100962.9		91720.9		96198.8	

The accession numbers of proteases: putative serine protease of *Glaciozymaantarctica *PI12 (CAQ76821), subtilisin-like protease *Pleurotus ostreatus* (CAG25549), pyrolysin *Laccaria bicolor* S238N-H82 (EDR11679), subtilisin-like protease *Phanerochaete chrysosporium * (CAG38357), subtilisin-like serine protease PR1C *Metarhizium anisopliae* var. *anisopliae* (CAD11898), serine endopeptidase *Hypocrea lixii* (CAL25578), peptidase *Pyrenophora tritici-repentis* (EDU50747), peptidase *Cryptococcus neoformans* var. *neoformans* JEC21 (AAW40780), subtilisin-like protease *Epichloe festucae* (ACB30121), subtilisin-like protease *Epichloe festucae* (ACB30119).
