# Supplementary material for: Molecular Cloning and Optimization for High Level Expression of Cold-Adapted Serine Protease from Antarctic Yeast Glaciozyma antarctica PI12
Source: Enzyme Res. 2014 Jun 30;2014:197938. doi: 10.1155/2014/197938 (PMC4100274; doi:10.1155/2014/197938)
Supplement: Supplementary file 1 — Genomic DNA sequence assembly of PI12 serine protease derived from DNA walking and genome sequence library (4081 bp) (5' to 3'). [file 197938.f1.docx]

**Supplementary figure**

**Genomic DNA sequence of cold-adapted PI12 serine protease (4081 bp) (5’-3’)**

Partial sequence from DNA Walking approach (1531 bp)

91531 bp)

TCACAGAAGTATGCCAAGCGAGGGGGGGGGGTCTGCTAGCGCGAATCGAGAAAACACAAAAGAACCTCCC

GACTTGGCGTTGCTTCTGCTCTTCTCCTTCTCCCAACCGCCGACAATAAAAACTATAACACACACACACC

CCATCTCGACGCCTGCAGACTTCACGACTCTCCTCACGCTCTTTACCGCCCCACGGGTCCCACCCACGAG

CCACG**ATG**CTCTTCCTCCCCGTCCTCCTCCTCCTCCTTCCCGGCGTCACTGCCTTCCTCAACCCAGTTAC

CAACCGCGCGACCAACGCCATCTCCTCGACTCAATACCTCTCTAACGCCTATATCCTCGAATTGGACCTC

TCCACCCCCGGCCTCGTCAAACGGGATAGCACGCCCGACTCTGTGCGTCCCGCGTTCCTCCCTCGCTGTC

GAAGACGAGCTAACAATGACGTGGTAGATCCTAGAGGACGTACTCACGTCCGTAGGCCGCAACGGTATCA

**Full genomic DNA sequence of cold-adapted PI12 serine protease (4081 bp)**

AGTACCAACTCCGCCACCGCTTTATCTCCCCGACTCTGTTCCACGGCGCTTCGATCACTGTCCCCCCTGG

AATCTCCCGCTCCCAAATCGCCTCTCTCCGCGGTATCAAAGTCCGTCCCTCTCTCTCCCCTACTCCCTCC

GTGCTAACCCTGCACGCAACAGCGCGTCTGGCCCGTTCGAAAGTTCTCCCGACCCAGCGCAGTAGTGGAC

GCCGATGGAGGAGGAAGCGGGTTCTCAGGGTCGCCTATCAAGGCGGCGCTCATGGGGGTGAAAGAGCTCG

GGAAGCGCGCGAACGCTTATGCTGGAGATACGTTTGGACCGCATGTCATGACGGGGGTTAATGAGACGCA

TGAGGCGGGGTTGTTGGGAGCTGGGATTAAGGTGTGTTTTCGTCTTTGTTTGGGGGAGGGGGGAGGGAGG

AGCTGATTAATGGGTGCAGATTGGGGTGCTGGACACTGGTGAGAAGCGGTTTGGAGGGAGGAGAGGATGG

AGCTGATGAGTGTGCAGGTGTTGATTATTTGAACCCGATTCTGGGAGGCTGCTTTGGACCTGGGTGCCAT

ATGTCGTTTGGGTACGACTTGGTTGGCGATGATTACGATGGAGATAACGCTCCTGTGCCGGATGTGGATC

CTTGTGCGTCCTTCCCTCCGCGATGGGCTGGAATCTGAGCTGACGGCGTTGTTGATCAGTTGCGAGCTGC

GATCCTCATGGTTCGTCTCGCTTTCCGCTCCTCTGATGCGCTCGCTGATGCACTTGGCAATAGGAACTCA

CGTTACGGGAATCATTGGAGCGCTCCCGAATGCGTTTGGATTTACTGGCGTCGCACCCGCCGCTACTCTG

GGCCACTACCGAGTGCGCTCCCTCTATCCGTTCTTCCTCGCAGCTCCCACTCACTCGTGCTTCCAGGTAT

TTGGCTGCACTGGCTTCGTCGGAGAAGATATCGTTCGTCCTCTCTCCAGTCCCGGCTAGCGATCCTGCTT

CTGACTTCGCATCTCCCTCTGAGATTCTCGCTGGACTCATGCGAGGAGTCGAGGACAACTGCAACGTCTT

GACCCTCTCTCTCGGAGGTCCAGGAGGGTGGGTCAAGGGCACGCCGGCGTCCATCCTTATCGACCAGATC

Partial sequence from recombinant plasmid (shotgun cloning) (1321 bp)

GAAGCGCAAGGCATTCTCGTCACCGTCGCCACTGGCAACTCGGGAGCTGAGGGTGAGCTCCTCGCTACTT

GCGCGTCGAGACCAGTACTAACAGGGGCGTGGACGCAGGCATGTTCTTCTCCGAGTCTCCCGCCTCGACG

ATCAACGGCCTTTCCATCGCATCCACGTCCGTCTCTCCCTTCCTTCGCACGCCACTCGCTCACACTCTCT

CGCAGGGACGTTACCGACCTCATCGCCTACAACGCCACCGTCTCAGGCCAACCTGCGATCCCTTACCTCT

CCGCCACGCCCCTCAACGTCGTCGCCAACAGCTTCCGCGTCCACTTCACCTCTACCGACCCCAACAACCC

CGTCGACGCCTGCTCTCCTCTTCCGGCTGGAGCGCCCGACTTCGCCAACTATGTTACGGTCGTTCAGCGT

GGGACTTGTACGTTCGTTACCAAGTACCAGAACGTTCTCAATGCTGGAGGGTGCGTCGGCTTCTTCTCTC

CCGACTGGATTGGGCGGAGACTGACCTTCTGAGCAGAAAGATCGTATTGTTGTACAACTCGGAGGGAGCT

GGGAACCTCCCTTACCTCACGCCCAACGGTGTCGGCATCGACGCCGTTGCAGGTCTTCGTCGTTCCGACG

GACTCAAGGTTCGTCTACGCTCTTCGGTGCTGAGGCCCTTCTCTGATCTCGCTCCGACAGCTTCTCTCGT

ACTATCAGAATGCCAACAAGCGTCTCACTCTGCGCTTCCCCAAGGGCAAGATCGTCGCAGGCTTGACCGA

TACCATCACCGGCGGACTCATCTCGTTCGTTCCTCTCCGCCTCCCTCGCTGGCCGATGAGCTGACGCGGT

CTTTGCAGGGGTTACTCGACGTTTGGTCCGACGAATGACCTCTACGGTCAGCCTACCCTCTCTGCCCCTG

GTGGCAACATCCTTTCGACCTTCCCTCTCTCCGAGGGAGGAGTGGCGGTCATCAGTGGGACGAGCATGTC

GTGCCCCTTTGTCGGTTCGTCCTTCTCCTCGTTCGTTCGCTGAGCGTCGACTGATCTTGCACGATCGCAG

CTGGATCTGCGGCGGTCCTCATGGCCGCTCGCGCTTCGGAGAACCTCACGCCGCTTGAGATCAGGAGTCT

CCTTACTACCACTGCGAAGCTTACGCCGGTCTCGCTCTTGGGATCGACGCCTTTGGTGAGCGTGATTCGT

CAAGGAGGAGGTACGTTCCTTTCTCGCTGCTTTGAGGATCGAGGCTGATTAGCGAGCTTCGCAGGACTCG

TTCAGGTTGCCAAGGCGCTCGCGGCCAAGACGCTAATCTCTCCTCACGAGCTCCTGCTCAACGACACTGC

GAACGCGAACTACGTCCAGACTATCAAGATCAAGAACACCAACTCGTGGGCGATGAAGTACACCTTCTCC

TCGGCCGTCGCCCAAGGACTCGGAACTTTCGACGCTTCGGGCGATATCCTCCCTACCCTCGACCCAGTCG

CCGTCTCTGGCGCACAGGCTACCGTCGCGTTCAACACTCGGATCCTCAGCGTCGCGCCCGGCGCGACGGG

GTCCGTCGTGGCGACTATCACGCCGCCGGTTCTTCCCGTAGCGGACGCTGCGAGGTTCCCTATCTTCTCT

GGGTGGATCAGGGTGAATGGGCAAGGCGCGAGGGATAGCAGTAGGAACGAGGCGTACACTGGTGCGTTTG

GCTCGCTTTTTGGGAGGTGCCGAGCTAATCGTTTCTCGTAGTCCCGTACTTTGGGCTTGCGGCGAAGATG

ATCGATATGCAAGGTGCGTCGATGGTTCTCTCGCTTCTCTCTCGGTTGCTCACTTCAAAGTGCAGTCCTC

GACACCACCGAGACCATTTACGGTCCGGGCTACGCCTACCCCTTCGTGATCGACGACGCGATTGGAGACA

TCCAATCCACCACAACGTCGTACTCCAGGAACCTCGGGCCCACCGTCTTCGCTCGCTTTGCCATGCGTCG

ACCGTCCTCATTTCCTGCGGCAGAGCGAGAGCTGATTCCGACCTCGAACAGCTGGAACCCTTCACTACAG

CCTCGATCTCGTCCTAGCCGACATCGCCTTTACCCCCACCTACCCCAACTCCTCCCCCGCCACTCGTTTC

GTCAAGCGCTCCCTCACGCAGCACACCTCTGCCGCTTCGCACCTCGCCAAGCGCCGCGTCTCCATCGCCA

CTATCAACCCCAAAGCCACCCTCGTCGCCGATCGACAGCTCCACTCGGACGTCCCTATCGAGGGCAACAT

CTTCACCCAACCCTTTACTGGAAGGGATTACCTCGTCGACGCAGCCCCGACGGGATCCACCGATCGTACC

GTCACTTTTAACGGGCAGTACGCCGAGAACGGCCTCGTGAGGACGGCTGTGACGGGGACTTCGTACCGCT

TCCTCCTTCGGGCGTTGAAGATCTCGGGAGACGCGATGTACGAGGATCAGTATGAGAGCTGGCTCTCGCT

ACCGTTCTCGTTCCGTGCG**TAG**

Sequence overlapping: Both partial sequences from recombinant plasmid 4Blunt/PI12 protease (shotgun cloning) and DNA walking were joined together and made up a total length of 2687 bp
